# Extension of the mammographic screening programme in Italy: cost-benefit analysis of early diagnosis in the 45–49 and 70–74 age groups

**DOI:** 10.3389/fmed.2026.1648666

**Published:** 2026-08-10

**Authors:** Eugenio Di Brino, Elena Maria Calosci, Michele Basile, Filippo Rumi, Debora Antonini

**Affiliations:** ALTEMS Advisory, Università Cattolica Del Sacro Cuore, Rome, Italy

**Keywords:** breast cancer prevention, cancer screening policies, cost-benefit analysis, early diagnosis, healthcare economics, mammographic screening, public health strategy

## Abstract

Extending Italy’s mammography screening programme to women aged 45–49 and 70–74 could markedly improve breast-cancer prevention, lowering mortality and optimizing healthcare resources. Using a decision-tree model based on national epidemiological data, we compared the current screening coverage scenario, characterized by a partial regional adoption, with a nationwide optimal coverage extension. Under the current screening coverage scenario, the screening programme covers 1,995,876 eligible women, with a per-patient cost of €9.52. The model estimates 55,663 annual incident cases across the total eligible women, with approximately 13,975 diagnosed at an early stage and 41,688 at an advanced stage. Average disease-management costs amount to €135.84 per patient, resulting in a total per-patient expenditure (screening plus management) of approximately €145.36. The optimal screening coverage scenario extends coverage to the full eligible population of 3,930,936 women, increasing the per-patient cost to €18.75. Extended screening shifts stage distribution toward earlier detection, with approximately 19,430 early-stage cases and 36,233 advanced-stage cases. Per-patient disease-management costs fall to €126.05, for a total per-patient expenditure of approximately €144.80. The near-perfect equivalence between the two scenarios confirms that the intervention is essentially resource-neutral: the additional screening expenditure is almost entirely offset by savings in disease-management costs driven by earlier diagnosis, while enabling the identification of approximately 5,454 additional early-stage cases. Overall, full national extension would require additional investment, almost entirely offsetting costs for the Italian National Health Service (NHS) while improving equity and long-term sustainability through wider access to early diagnosis. Strategies to increase adherence, reduce regional inequalities, and integrate innovative technologies remain crucial, and inclusion of the programme in the Essential Levels of Care (LEA) would guarantee long-term equity and sustainability beyond cost savings.

## Introduction

Breast cancer (BC) is one of the most common forms of cancer among women and is one of the leading causes of cancer death in this population. In Italy, 53,060 new cases were expected in 2024, accounting for 59.3% of all female cancers (1). BC is characterized by the uncontrolled growth of malignant cells in breast tissue, usually starting in the ducts or lobules of the breast. BC is often asymptomatic; furthermore, the risk of developing it is estimated at around 11–12% over the course of a woman’s life (2). Major risk factors include age, family history, hereditary genetic mutations (BRCA1 and BRCA2), prolonged exposure to oestrogen and unhealthy lifestyles (poor diet, alcohol, obesity, sedentary lifestyle) (2). Early diagnosis and therapeutic advances have substantially improved the prognosis of the disease, although mortality remains high in cases of late diagnosis or particularly aggressive variants (3). In this context, the introduction of systematic mammography screening has enabled the detection of BC at earlier stages and significantly increasing the likelihood of cure: available evidence estimates a 20–30% reduction in mortality among women who participate in screening programmes (4). The diagnostic process relies on clinical assessment combined with imaging techniques, primarily mammography and ultrasound, with magnetic resonance imaging (MRI) reserved for selected cases. Histological confirmation is obtained through biopsy. Therapeutic management is guided by tumor type and stage and may include surgery, radiotherapy, systemic treatments, and targeted therapies for HER2-positive disease (5). Currently, the screening programme in Italy is mainly aimed at women aged between 50 and 69, but recent studies suggest that early diagnosis in younger women, starting at age 45, could increase the chances of detecting tumors at an early stage, when treatment options are less invasive and the chances of recovery are higher. In this age group, although the incidence of BC is lower than in older women, tumors tend to be more aggressive, making early screening a crucial opportunity to improve prognosis (6). Similarly, including women up to the age of 74 could allow for the detection of tumors that develop later in life and which, without regular screening, could be diagnosed at an advanced stage, when treatment options are more limited and less effective. Extending the screening campaign to these age groups can significantly reduce the costs associated with treatments for advanced tumors, which require complex and expensive interventions such as chemotherapy, extensive surgery and intensive radiotherapy. Moreover, earlier detection may improve patients’ quality of life by reducing the need for aggressive treatments and helping to preserve both physical and psychological well-being (6). Both these age ranges have specific benefits and potential harms of population-based screening in women at risk: below the age of 45, lower incidence and higher rates of false positives and overdiagnosis may reduce the overall value of screening, while beyond the age of 74 the benefits decrease due to competing mortality risk, comorbidities, and reduced life expectancy.

In the Italian healthcare setting, evidence regarding the economic benefits of extending the eligibility criteria for organized mammography screening remains limited, representing an important gap in informing healthcare policy decisions. This study aims to assess the economic consequences of extending the screening programme through the use of a Cost–Benefit Analysis (CBA) with particular attention to the potential reduction in costs associated with advanced-stage cancer diagnoses avoided through screening.

## Policy options and implications

The CBA expresses both costs and consequences in monetary units, enabling a direct comparison of the net economic impact of the intervention (7). In this context, benefits were operationalized as avoided disease-management costs resulting from earlier-stage diagnosis attributable to screening. The analysis was built using epidemiological and clinical data from national cancer registries and published studies, representative of the Italian healthcare context (8, 9). The analysis adopts the payer’s perspective (Italian National Health System, NHS), including only direct healthcare costs while excluding indirect costs such as productivity losses or social impacts. A 1-year time horizon was adopted, consistent with a single screening round, and no discounting was applied.

**FIGURE 1:**
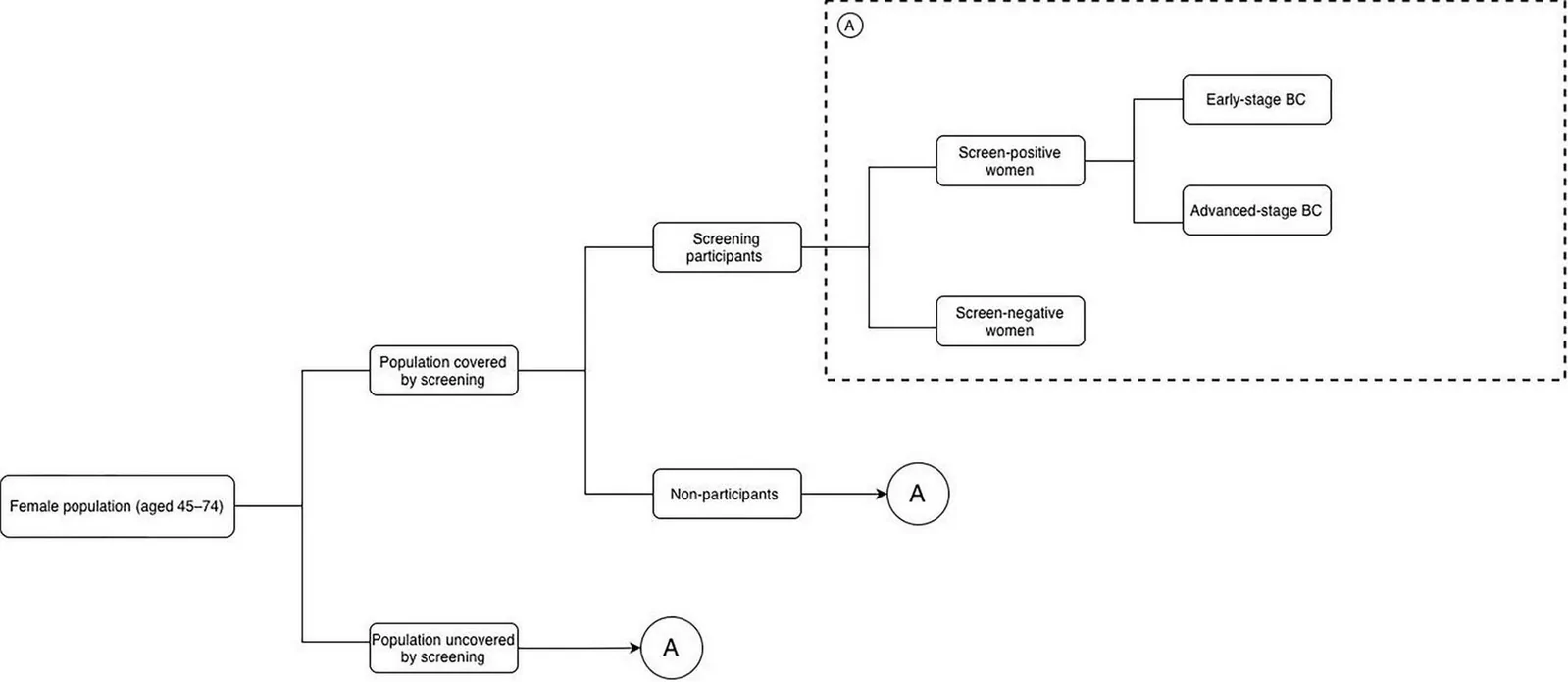
Graphical representation of the decision-tree structure of the breast cancer screening model in Italy.

The analysis is based on a decision tree–based economic model (Figure 1) that reproduces diagnostic pathways by applying observed screening participation rates and assumptions on the stage distribution of the disease. All transition probabilities used in the model are reported in Table 1 (9, 10). This approach makes it possible to estimate the economic impact of the shift from advanced-stage to early-stage diagnoses. Two scenarios were compared in the CBA: the current coverage scenario, reflecting the existing level of screening implementation, and the optimal coverage scenario, which considers a universal screening programme for women aged 45–74.

**TABLE 1:** Transition probabilities (9, 10).

Parameters	Value (%)
Coverage and Adherence	
Screening adherence rate	53.80%
Women adhering to screening (% of total eligible population)	53.80%
Women not adhering to screening (% of total eligible population)	46.20%
Probability of BC diagnosis by stage – Without screening	
Early-stage diagnosis	15.00%
Advanced-stage diagnosis	85.00%
Probability of BC diagnosis by stage – With screening	
Early-stage diagnosis	52.00%
Advanced-stage diagnosis	48.00%

The population eligible for the extension of the mammography screening programme was quantified by integrating regional screening coverage (9) with updated demographic data from ISTAT (2024) (11), focusing on women in the age groups currently uncovered by organized screening (45–49 and 70–74). Table 2 provides a regional overview of the eligible population stratified by age group: out of 3,930,936 women, only 50.77% are currently covered by organized screening programmes. Accordingly, the current screening coverage scenario includes 1,995,876 women, with 1,935,060 uncovered.

**TABLE 2:** Quantification of the population eligible for screening (9, 11).

	Uncovered population (n)		Covered population (n)	
	Age range 45–49 y	Age range 70–74 y	Age range 45–49 y	Age range 70–74 y
Abruzzo	46,256	-	-	40,029
Basilicata	18,940	16,646	-	-
Calabria	-	-	65,742	55,304
Campania	145,384	-	61,700	150,282
Emilia-Romagna	170,369	127,376	-	-
Friuli-Venezia Giulia	43,154	-	-	38,139
Lazio	-	162,456	226,515	-
Liguria	-	49,212	52,131	-
Lombardy	374,180	281,273	-	-
Marche	55,536	45,439	-	-
Molise	-	-	10,045	9,480
Piedmont	-	-	154,709	133,469
Apulia	-	-	147,772	118,019
Sardinia	-	-	60,833	52,190
Sicily	-	-	174,062	141,830
Tuscany	139,540	110,943	-	-
Trentino-Alto Adige	19,474	15,089	18,311	12,613
Umbria	-	26,844	32,138	-
Aosta Valley	4,534	3,732	-	-
Veneto	-	139,499	179,747	-
Total population uncovered by age group	1,183,705	751,355	-	-
Total Population uncovered	-	1,935,060	-	-
Total population covered by age group	-	-	1,017,367	978,509
Total population covered				1,995,876

According to the Italian NHS perspective, unitary screening costs were based on the Ministry of Health tariff schedule (12), with mammography set at €34.85; for the purposes of this analysis, the per-patient screening cost was set at this tariff (12). Costs associated with disease management were derived from a real-world analysis of the burden of BC in Italy based on administrative healthcare databases (13). Annual per-patient costs were estimated at €4,226 for early-stage BC and €11,380 for advanced/metastatic disease (13).

A deterministic one-way sensitivity analysis (one-way DSA) was conducted to assess model robustness by varying each key parameter within a ± 25% range. The following parameters were included in the analysis: covered target population, uncovered target population, screening adherence rate, incidence rate, early-stage diagnosis rate with screening, early-stage diagnosis rate without screening, mammography screening cost, early-stage breast cancer management cost, and advanced-stage breast cancer management cost.

Across the full population of 3,930,936 women, the model estimates approximately 55,663 annual incident BC cases (incidence rate: 1.42%). In the current screening coverage scenario, 13,975 cases (25.1%) are diagnosed at an early stage and 41,688 (74.9%) at an advanced stage. In the optimal screening coverage scenario, extended screening coverage shifts this distribution to 19,430 early-stage cases (34.9%) and 36,233 advanced-stage cases (65.1%), representing an incremental gain of approximately 5,454 additional early-stage diagnoses. Extending the mammography screening programme entails higher per-patient screening costs (€18.75 in the optimal screening coverage scenario versus €9.52 in the current screening coverage scenario) but simultaneously enables improved disease management through greater detection of tumors at an early stage. Disease-management costs per patient amount to €135.84 in the current screening coverage scenario and decrease to €126.05 in the optimal screening coverage scenario, reflecting the shift toward earlier detection driven by broader screening coverage. When total per-patient expenditure is considered, combining screening and disease-management costs, the two scenarios converge to virtually identical figures: €145.36 in the current screening coverage scenario and €144.80 in the optimal screening coverage scenario. This near-perfect equivalence confirms that the extension of the screening programme is substantially resource-neutral from the perspective of the NHS.

**FIGURE 2:**
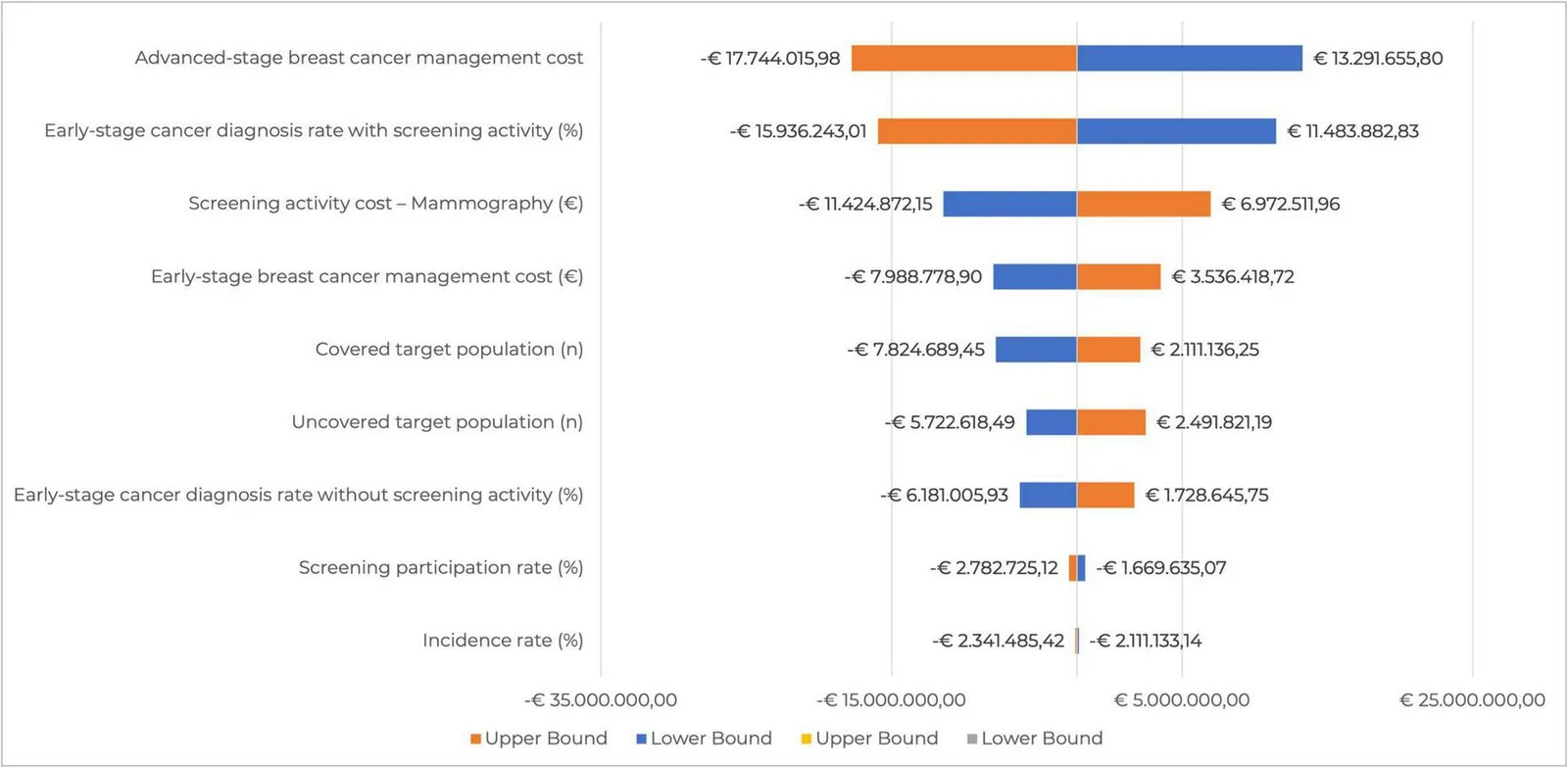
Tornado diagram of the one-way deterministic sensitivity analysis.

The net annual cost impact was graphically illustrated in a tornado chart (Figure 2). The parameters with the greatest influence on the results were the cost of managing advanced-stage BC, with estimated changes in the total saving ranging from approximately –€17.7 million to +€13.3 million, and the early-stage diagnosis rate with screening, ranging from approximately –€15.9 million to +€11.5 million. The cost of mammography screening and the cost of managing early-stage disease also showed a relevant impact. Among the remaining parameters, the early-stage diagnosis rate without screening and population size showed a moderate effect, while the adherence rate and the incidence rate had the smallest influence on the total saving estimate. Overall, the DSA results confirm the robustness of the model and its sensitivity primarily to clinical and cost parameters related to disease stage, while demographic parameters have a more limited influence on the total saving estimate.

These estimates are strengthened by their reliance on national cancer registries, ISTAT demographic data, and Italian tariff and real-world cost sources, with robustness confirmed by the one-way sensitivity analysis, whose results were driven mainly by clinical and cost parameters rather than demographic assumptions. Two limitations should nonetheless be noted. First, the stage distribution assumed for unscreened women (15% early-stage, 85% advanced-stage) is a modelling assumption calibrated to a resource-neutral outcome rather than an empirically observed value, and downstream costs such as long-term follow-up, hospitalization, and end-of-life care were not modelled, suggesting that the true savings are likely underestimated. Second, the 1-year horizon and payer perspective exclude the longer-term survival, quality-of-life, and productivity benefits that a lifetime cost-utility framework would capture; future research should extend this analysis toward a probabilistic, lifetime cost-utility model incorporating QALYs and indirect costs.

## Actionable recommendations

The CBA confirms that extending the mammography screening programme in Italy to women aged 45–49 and 70–74 represents an effective and economically sustainable strategy. Early diagnosis improves disease management, reducing the number of advanced-stage cases and associated treatment costs. To maximize the impact of the programme extension in Italy, several policy measures are recommended:

- **Inclusion in Essential Levels of Care (LEA):** Extend economic coverage of mammography screening to women aged 45–74, ensuring nationwide access.
- **Improving participation:** Increase adherence through targeted awareness campaigns involving patient associations and healthcare professionals, active and personalized invitations, digital reminders, and expanded access via pharmacies and local health centers. Incentives such as paid leave for women attending screening could further boost participation.
- **Reducing regional disparities:** Monitor programme coverage and quality across regions to ensure equitable access. Because the intervention is broadly cost-neutral at the system level, its inclusion within the LEA would not represent a new structural cost for the NHS, but rather a redistribution of existing expenditure toward earlier, less resource-intensive stages of disease. Formal inclusion in the LEA would also address the marked regional heterogeneity documented in Table 2, where only about half of eligible women are currently covered and access to the 45–49 and 70–74 age groups varies substantially across regions.
- **Leveraging technology and AI:** Develop AI-based tools to improve diagnostic accuracy and reduce false positives and integrate screening results into the Electronic Health Record to optimize patient follow-up.

Implementing these measures would maximize the health benefits of the screening extension, promote equity, optimize healthcare resources, and improve clinical outcomes for thousands of women. While short-term cost savings may vary according to demographic characteristics and regional incidence patterns, the extended programme contributes to improved early detection, reduced mortality, and a more equitable distribution of preventive healthcare services nationwide. These recommendations align with the National Cancer Plan 2023–2027 (14), which aims to strengthen interregional cooperation, expand digital reporting systems, increase public awareness, and promote broader adoption of extended screening, reinforcing Italy’s commitment to evidence-based, inclusive BC prevention.

International studies, although based on different methodological frameworks including cost-effectiveness and cost-utility analyses, consistently support the extension of mammography screening to broader age groups. The UK Age Trial, launched in 1991 and involving over 160,000 women, demonstrated that annual screening starting at age 40 reduced BC mortality, influencing national screening policies. Economic evaluations in the UK estimated a cost of £27,400 per Quality-Adjusted Life Year (QALY) gained for extending the target age, with a 29% probability of being cost-effective at the £30,000 threshold (15). In the United States, the USPSTF recommends biennial screening for women aged 40–74, citing a moderate net benefit, while evidence for women over 75 remains insufficient (16). Similarly, a 2024 Canadian cost-effectiveness analysis reported a cost of $1,447 per QALY gained for annual screening of women aged 40–74 compared with biennial screening of women aged 50–74 (17), and a Finnish study positively evaluated the extension of biennial screening to women aged 45–74 compared with the existing national strategy (18). Taken together, both the Italian analysis and international evidence confirm that extending mammography screening to women aged 45–74 is clinically justified and economically sustainable.

## Conclusion

The expansion of the mammography screening programme in Italy represents a fundamental strategy for strengthening prevention and promoting early diagnosis of BC. This analysis demonstrates that extending the programme to women aged 45–74 is economically sustainable and substantially resource-neutral: the additional screening costs are almost entirely offset by savings in disease-management costs from earlier-stage diagnosis, delivering a meaningful gain in early diagnosis at essentially no net cost. Extending the programme nationwide would ensure more equitable access to timely diagnosis and reduce current regional inequalities in prevention, reframing the question from whether Italy can afford this extension to whether it can justify forgoing a resource-neutral opportunity to improve outcomes and equity. For this strategy to be effective, uniformity in access, reimbursement, and adherence across all regions is essential.
